# Global Analysis of Cellular Protein Flux Quantifies the Selectivity of Basal Autophagy

**DOI:** 10.1016/j.celrep.2016.02.040

**Published:** 2016-03-03

**Authors:** Tian Zhang, Shichen Shen, Jun Qu, Sina Ghaemmaghami

**Affiliations:** 1 Department of Biology, University of Rochester, Rochester, NY 14627, USA; 2 Department of Pharmaceutical Sciences, University at Buffalo, Buffalo, NY 14214, USA; 3 Department of Biochemistry, Jacobs School of Medicine and Biomedical Sciences, University at Buffalo, Buffalo, NY 14214, USA; 4 New York State Center of Excellence in Bioinformatics and Life Sciences, Buffalo, NY 14203, USA

## Abstract

In eukaryotic cells, macroautophagy is a catabolic pathway implicated in the degradation of long-lived proteins and damaged organelles. Although it has been demonstrated that macroautophagy can selectively degrade specific targets, its contribution to the basal turnover of cellular proteins has not been quantified on proteome-wide scales. In this study, we created autophagy-deficient primary human fibroblasts and quantified the resulting changes in basal degradative flux by dynamic proteomics. Our results provide a global comparison of protein half-lives between wild-type and autophagy-deficient cells. The data indicate that in quiescent fibroblasts, macroautophagy contributes to the basal turnover of a substantial fraction of the proteome at varying levels. As contrasting examples, we demonstrate that the proteasome and CCT/TRiC chaperonin are robust substrates of basal autophagy, whereas the ribosome is largely protected under basal conditions. This selectivity may establish a proteostatic feedback mechanism that stabilizes the proteasome and CCT/TRiC when autophagy is inhibited.

## Graphical abstract

**In Brief:**

Macroautophagy is a catabolic pathway for the degradation of proteins in eukaryotic cells. Zhang et al. quantified the relative contribution of macroautophagy to basal proteome turnover by comparing protein half-lives between wild-type and autophagy-deficient fibroblasts. The data provide a global map of the selectivity of macroautophagy in human cells.

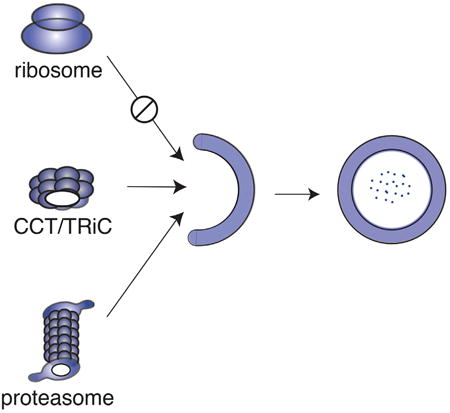

## Introduction

Within a cell, proteins are in a state of dynamic equilibrium and are continuously synthesized and degraded (Goldberg and St John 1976). Protein half-lives are highly variable and can range from a few minutes (e.g., the tumor suppressor p53) to several years (e.g., myelin basic protein) (Claydon and Beynon, 2012; Levine, 1997; Price et al., 2010; Sabri et al., 1974). The half-lives of proteins are often intimately linked to their functions, and disruptions in protein degradation have been associated with a number of pathological conditions (Nedelsky et al., 2008). The constitutive degradation rate of a protein is established by multiple substrate-specific and nonselective cellular pathways (Goldberg and St John, 1976). Within the cytoplasm of eukaryotic cells, the two primary pathways for protein degradation are the ubiquitin proteasome system (UPS) and the macroautophagy pathway (for simplicity, hereafter referred to as “autophagy”) (Mizushima and Komatsu, 2011). It is generally believed that the UPS is responsible for the degradation of transient shortlived proteins, while autophagy contributes to the degradation of stable long-lived proteins (Ciechanover, 2005). However, beyond these general trends, the relative contributions of individual pathways to protein degradative flux have not been quantified on proteome-wide scales. In this study, we quantified the relative contribution of autophagy to proteome turnover by comparing protein half-lives between wild-type and autophagy-deficient cells.

The process of autophagy involves the sequestration of cytoplasmic proteins and organelles by double-membraned autophagosomes and their subsequent fusion and degradation by lysosomes (Klionsky and Emr, 2000). Autophagy is constitutively active at low levels under nutrient-rich (basal) conditions and is upregulated during periods of starvation (Mizushima, 2007b). Starvation-induced autophagy is a critical nutritional response designed to replenish cellular amino acid supplies (Singh and Cuervo, 2011). Basal autophagy, on the other hand, is thought to be responsible for constitutive turnover of a subset of proteins and clearance of damaged proteins and organelles (Fimia et al., 2013). The functional importance of autophagy has been demonstrated in a number of eukaryotic model organisms. For example, systemic inhibition of autophagy in mice results in neonatal lethality (Kuma et al., 2004). Targeted tissue-specific inhibition of autophagy has been shown to disrupt protein homeostasis as evidenced by accumulation of ubiquitinated protein aggregates over time (Hara et al., 2006). These proteostatic disruptions have a number of deleterious tissue-specific phenotypes. For example, suppression of autophagy in neurons leads to extensive neurodegeneration, and liver-specific inhibition of autophagy leads to the development of hepatic adenomas (Takamura et al., 2011). Defects in the autophagy pathway have also been linked to a number of neurodegenerative disorders (Nixon, 2013).

Historically, autophagy has been thought to be a bulk degradation pathway that non-selectively sequesters portions of the cytoplasm for lysosomal clearance (Mizushima, 2007a). However, recent data have highlighted the ability of autophagy to selectively eliminate specific substrates, including mature ribosomes, peroxisomes, endoplasmic reticulum, and mitochondria (Fimia et al., 2013). Selective modes of autophagy are thought to play an important quality control function by targeting damaged proteins and dysfunctional organelles for degradation. The detailed molecular mechanisms of cargo selection and regulation of selective autophagy remain under active investigation. In general, these pathways appear to rely on specific cargo-recognizing receptors (e.g., SQSTM1/p62, NBR1, and NIX) that act as linkers between target cytoplasmic components and proteins embedded in autophagosome membranes (e.g., LC3) (Rogov et al., 2014). This repertoire of receptors provides potential mechanisms of cargo selection for diverse substrates. However, it remains unclear what subset of the proteome can be targeted by basal autophagy and to what extent basal autophagy discriminates between different protein substrates.

Traditionally, the kinetics of degradative flux has been studied by monitoring the incorporation and removal of radioactive tracer amino acids into bulk proteins in “pulse-chase” experiments (Bjørkøy et al., 2009; Buchanan, 1961; Garfinkel and Lajtha, 1963). Recent advances in mass spectrometry have enabled global analyses of protein degradation by time-resolved stable isotope labeling (Claydon and Beynon, 2012). In this study, we have used clustered regularly interspaced short palindromic repeats (CRISPR) (Cong et al., 2013; Horvath and Barrangou, 2010) to create autophagy-deficient (*ATG5*^−/^*^−^* and *ATG7*^−/^*^−^*) primary human fibroblasts, and then utilized time-resolved isotopic labeling and mass spectrometry to provide a quantitative global comparison of basal protein degradation rates between these and wild-type cells.

## Results

### Creation and Characterization of Autophagy-Deficient Primary Human Fibroblasts

In orderto establish autophagy-deficient primary cell models, we used CRISPR (Cong et al., 2013; Horvath and Barrangou, 2010) to knock out *ATG5* and *ATG7* in human diploid fibroblasts expressing the catalytic component of human telomerase (HCA2-hTert) (Bodnar et al., 1998; Voskarides and Deltas, 2009). HCA2-hTert fibroblasts can be continuously propagated without becoming senescent yet display the hallmarks of primary cells including serum-independent growth control and a stable diploid karyotype (Bodnar et al., 1998; Vaziri and Benchimol, 1998). They exhibit robust contact inhibition and can be maintained in a quiescent state for extended periods of time while retaining the ability to re-enter the cell cycle. In their quiescent state, fibroblasts are metabolically active and autophagy occurs at elevated basal levels (Lemons et al., 2010; Valentin and Yang, 2008). The ability to experimentally maintain HCA2-hTert cells in a quiescent state enables long-term isotopic labeling experiments in viable non-dividing cells. In such a state, the extent of fractional isotopic labeling for a protein is solely dictated by protein degradation and is not influenced by diluting effects of cellular proliferation (Claydon and Beynon, 2012; Edenetal., 2011). Thus, unlike dividing cell lines, analysis of fractional labeling in quiescent cells can provide accurate measurements of degradation kinetics of long-lived proteins with half-lives significantly longer than the doubling time of the cell.

**Figure 1:**
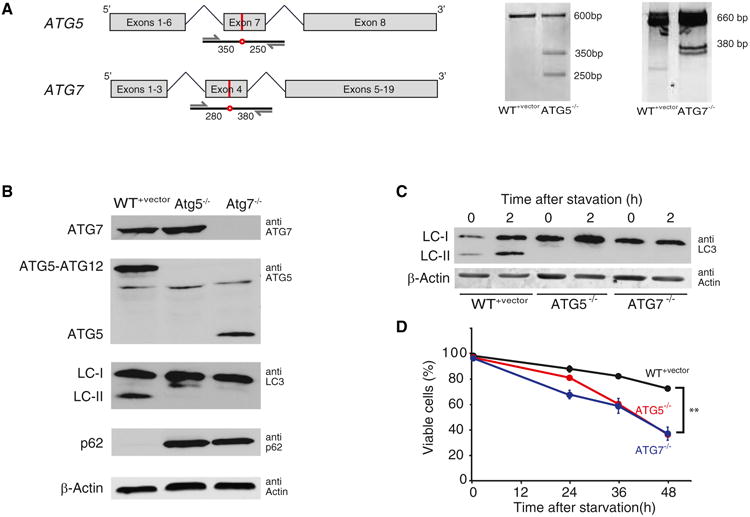
Creation and Validation of Autophagy-Deficient Human Fibroblasts (A) The schematics on the left are gene maps of human *ATG5* and *ATG7* showing the site of the introduced mutations (red line and circle) and the PCR strategy for amplifying the mutated genomic regions for the SURVEYOR assay. The numbers indicate the expected sizes of the mutated amplicon fragments after SURVEYOR nuclease cleavage. The right panel shows the results of the SURVEYOR assay indicating the introduction of mutations at expected genomic positions. (B)Western blots show the expression of ATG5, ATG7, LC3, p62, and β-actin control in WT^+vector^,*ATG5*^−/^*^−^,* and *ATG7*^−/^*^−^* cells. The blots indicate that ATG5 and ATG7 are completely knocked out in HCA2-hTert cells. The deletion of ATG5 and ATG7 results in complete disappearance of LC3-II, and the deletion of ATG7 prevents the conjugation of ATG5 to ATG12. The basal level of p62 is significantly elevated in *ATG5*^−/^*^−^* and *ATG7*^−/^*^−^* cells. (C) LC3-II levels increase under starvation conditions in WT^+vector^, but not *ATG5*^−/^*^−^* and *ATG7*^−/^*^−^* cells. (D) *ATG5*^−/^*^−^* and *ATG7*^−/^*^−^* cells are more sensitive to amino acid starvation. Cell viabilities were measured after different periods of amino acid starvation. Each time point was measured in three replicate experiments, and the error bar indicates SD. n = 3 biological replicates; **p < 0.01. See also Figure S1.

In order to create *ATG5*^−/^*^−^* and *ATG7*^−/^*^−^* HCA2-hTert fibroblasts using CRISPR, five single-guide RNAs (sgRNAs) targeting three different exons in each gene were designed. We used a lentiviral CRISPR expression system where the mammalian codon-optimized Cas9 nuclease was co-expressed with each sgRNA (Shalem et al., 2014). The cloned lenti-CRISPR vectors were used for viral production and human HCA2-hTert fibroblasts were infected with the resulting lentiviruses. Using the SURVEYOR assay (Voskarides and Deltas, 2009; Ran et al., 2013), we showed that sgRNAs targeting exon 7 of *ATG5,* and exon 4 of *ATG7* succeeded in introducing mutations in the intended genes (Figure 1A). Mutant isogenic clones were isolated by limiting dilution and each clone was shown to contain frameshift insertion-deletion (*indel*) mutations in the respective targeted gene (Figure S1). The complete knockout of ATG5 and ATG7 at the protein level was verified by western blots (Figure 1B).

ATG5 and ATG7 are part of a dual protein conjugation system that is required for the formation of autophagosomes (Komatsu et al., 2005; Mizushima et al., 2001). The conjugation of ATG5 to ATG12 requires ATG7, and both ATG7 and the ATG5-ATG12 complex are required for the conjugation of phosphate-dylethanolamine to LC3 (the mammalian homolog of Atg8) and its localization to the membrane of the developing autophagosome (Ichimura et al., 2000; Suzuki et al., 2001). The conversion of LC3 from an unlipidated form (LC3-I) to a lipidated form (LC3-II) and its subsequent localization to the autophagosome are required for membrane expansion and formation of mature autophagosomes (Nakatogawa et al., 2007). The expected disruptions in these two conjugation systems are evident in the mutant clones. The deletion of ATG5 and ATG7 results in complete disappearance of LC3-II, and the deletion of ATG7 prevents the conjugation of ATG5 to ATG12 (Figure 1B).

p62 (also known as SQSTM1) is a scaffold protein commonly found in ubiquitinated inclusion bodies (Ciuffa et al., 2015). p62 has been shown to play an important role as a receptor in some selective modes of autophagy by targeting specific substrates to autophagosomes. p62 is itself constitutively degraded by autophagy, and its expression level is a marker of autophagic flux (Bjørkøy et al., 2009). As expected, the basal level of p62 is significantly elevated *inATG5*^−/^*^−^* and *ATG7*^−/^*^−^* cells (Figure 1B). Together, the above data confirm that autophagy is constitutively active at basal levels in quiescent HCA2-hTert cells and that this activity is fully abolished in *ATG5*^−/^*^−^* and *ATG7*^−/^*^−^* clones.

*ATG5*^−/^*^−^* and *ATG7*^−/^*^−^* cells can readily proliferate in rich media supplemented with the full complement of amino acids. Under starvation conditions, wild-type cells upregulate autophagy as indicated by increased levels of LC3-II detected by western blots (Figure 1C). This marker of starvation-induced autophagy is completely absent in the mutant clones. Concomitantly, in comparison to wild-type cells, mutant cells experience a faster rate of cell death following the initiation of amino acid starvation (Figure 1D).

### Global Analysis of Protein Degradation Rates

**Figure 2:**
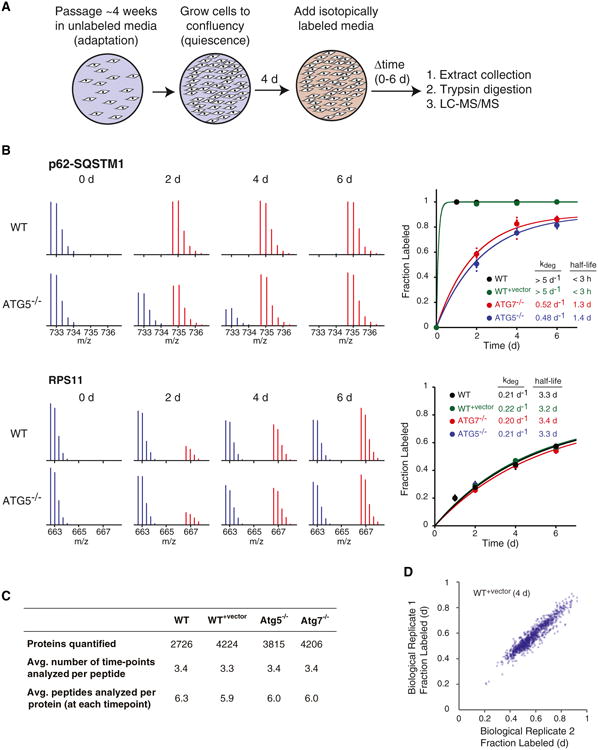
Global Measurement of Degradation Rates by Isotopic Labeling and LC-MS/MS (A) The schematic illustrates the experimental design. Cells were cultured in unlabeled (^12^C) media. Four days after reaching confluency, labeled (^13^C) media was added to the cells. During the following 6 days, cells were collected at different time points for LC-MS/MS analysis. (B) Isotopic labeling kinetics of p62-SQSTM1 and RPS11 are shown as example proteins. The spectra show the increase in fractional isotopic labeling of single peptides mapped to the two proteins. The unlabeled (“light”) spectra are shown in blue and the labeled (“heavy”) spectra are shown in red. Data from all peptides mapped to the proteins were combined and the kinetics of labeling were fitted to a first-order exponential equation to measure the degradation rate constant (*k_deg_*) and half-life of each protein in the four genetic backgrounds. The scatterplots indicate the median of peptide measurements at different time points, and the error bars indicate the SD of all peptides mapped to each protein. (C) Numbers of peptides and proteins detected and quantified in the proteomic analyses. (D)Scatterplot indicating a comparison of measured fractional labeling of WT^+vector^ proteins at 4 days for two biologically replicate experiments. See also Figures S2–S4.

It had previously been shown that time-resolved analysis of fractional isotopic labeling could be used to accurately measure protein degradation rate constants (and hence half-lives) on proteome-wide scales (Cambridge et al., 2011; Claydon and Beynon, 2012; Price et al., 2010; Zhang et al., 2014). This approach uses mass spectrometry and stable isotope labeling with amino acids in cell culture (SILAC) (Ong et al., 2002; Zhang et al., 2014) to detect and quantify the incorporation kinetics of isotopically labeled amino acids in proteins (Figure 2A). Here, we used this technology to determine the proteome-wide impact of autophagy inhibition on protein degradation rates.

Wild-type cells (WT), wild-type cells transfected with lenti-CRISPR vector lacking an sgRNA sequence (WT^+vector^), and *ATG5*^−/^*^−^* and *ATG7*^−/^*^−^* cells were grown to confluency. The quiescence states of cultures were verified by analysis of DNA content with flow cytometry and upregulation of the cyclin-dependent kinase inhibitor p27 (Coats et al., 1996) (Figure S2). After reaching quiescence, the cultures were switched to a media containing ^13^C-labeled lysines and arginines. The cells were collected after 0, 2, 4 and 6 days of labeling. Cell extracts were digested with trypsin using a surfactant-aided precipitation/on pellet-digestion (SOD) procedure that provides high peptide recovery from both soluble and membrane proteins (An et al., 2015; Duan et al., 2009). The digested peptide samples were resolved on a nano liquid chromatography (LC) column prior to analysis by an Orbitrap Fusion Tribrid mass spectrometer (See Experimental Procedures for additional details). Peptides were identified from tandem mass spectrometry (MS/MS) spectra by searching against the *Homo sapiens* Swiss-Prot database using the integrated Andromeda search engine with the software MaxQuant (Cox et al., 2011), and the relative ratio of labeled and unlabeled peptide MS1 spectra were quantified. Searches against decoy sequences indicated that the false discovery rates for protein identifications were less than 1% for all experiments. For each peptide, the relative labeling ratio was determined by a regression model fitted to all scans in a given elution peak.

Labeling ratios for proteins were determined as the median of all peptides assigned to each protein. Measurements of fractional labeling at different time points were fitted to a single exponential function to obtain first order degradation rate constants (*k_deg_*) for each protein. The formal kinetic model relating fractional labeling to *k_deg_* is presented in Experimental Procedures. The kinetic analysis for two proteins, p62 and the 40S ribosomal protein RPS11, is shown in Figure 2B as illustrative examples. Analyses of labeling kinetics of multiple peptides mapped to each protein provide measurements of degradation rate constants and half-lives. As expected, the degradation rate of the autophagy receptor p62, a known substrate of autophagy, was significantly reduced in autophagy-impaired cells from >5 d^−1^ to ∼0.5 d^−1^. Conversely, the degradation rate of RPS11 was largely unaffected and remained at ∼0.2 d^−1^ in the presence or absence of functional autophagy.

**Figure 3:**
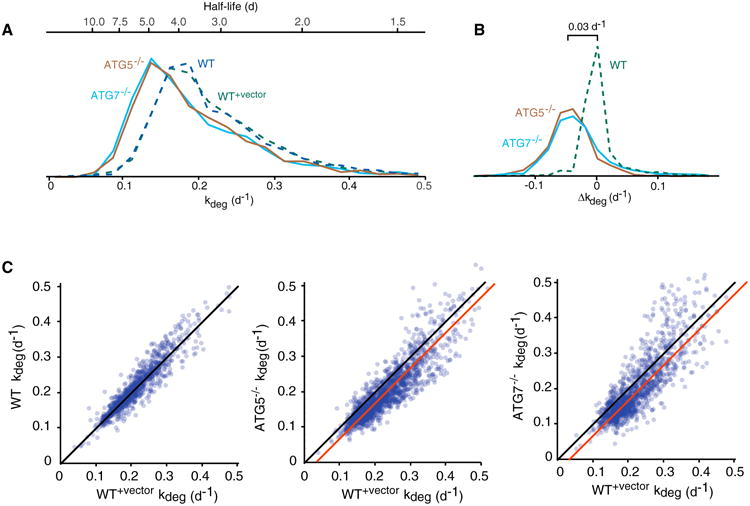
Global Impact of Autophagy Impairment on Basal Degradation Rates (A) Distribution of protein half-lives and degradation rates in WT, WT^+^*^vecto^*^r^, *ATG5*^−/^*^−^,* and *ATG7*^−/^*^−^* cells. (B)Distribution of differences in degradation rates (Δ*k_deg_*) between WT^+vector^ cells, and *ATG5*^−/^*^−^, ATG7*^−/^*^−^,* and WT cells. (C) Pairwise comparisons of protein degradation rates in WT, WT^+vector^, *ATG5*^−/^*^−^,* and *ATG7*^−/^*^−^* cells. Black lines indicate identity lines, and red lines indicate best-fit lines to the proteome-wide data.

In total, we were able to quantify the labeling kinetics of 2,726, 4,224, 3,815, and 4,206 protein groups in WT^−vector^, WT^+vector^, *Atg5*^−/−^ and *Atg7*^−/−^ cells, which together comprise ∼5,500 distinct protein groups (Figure 2C; Tables S4, S5, and S6). The higher number of analyzed proteins for the latter three backgrounds reflects additional fractionation steps of cell extracts that were conducted prior to the LC-MS/MS analysis in order to provide deeper proteome coverage (see Experimental Procedures for additional details). For most proteins, multiple independent time-points were included in the analysis. On average, approximately six independent peptides were analyzed to determine the degradation rate of each protein. For all subsequent analyses, only proteins that were quantified by more than one peptide analyzed in more than one time-point were considered. We also excluded rapidly degrading proteins that were completely labeled at the earliest time-point, for which exact degradation rates could not be measured. Three observations attest to the robustness of the data and computational analysis. First, there is little variation in measurements of fractional labeling at a time-point obtained from biological replicates (Pearson correlation coefficient = 0.95, Figure 2D). Second, there is little variation in protein rate measurements obtained from WT and WT^+vector^ cells (Figure 3). Third, there is relatively little variability in the labeling kinetics of multiple peptides mapped to the same protein. The median coefficient of variation (CV) in degradation rate measurements between peptides encompassing a single protein is significantly less than the CV among all peptides for a given genetic background (Figure S3).

In equating rate constants for fractional labeling to degradation rate constants, two important criteria must be met (Guan et al., 2011). First, the cells must be in a non-proliferating state and cellular protein levels must remain at a steady-state level throughout the labeling time course. Second, the cellular pool of precursor amino acids must become fully labeled prior to the first time point and remain fully labeled during the entire experiment (Zhang et al., 2014). In our quiescent cultures, cells are in a non-proliferating state and total protein levels do not change during the course of the experiment (Figure S4A). Furthermore, using isotopomer analysis (Hellerstein and Neese, 1992), we showed that when exposed to fully ^15^N-labeled media (where all 20 natural amino acids are isotopically labeled), the fraction of proteins that are synthesized within the first day is almost fully labeled. Thus, the pool of precursor amino acids utilized for protein synthesis is completely labeled priorto the first measured time point, satisfying a key assumption in our analysis (Figure S4B).

For the subset of proteins that were quantifiable by our analysis, half-lives generally ranged from a few hours to several days (*k_deg_* ranged from 0.05 d^−1^ to 0.5 d^−1^). Distributions and pairwise comparisons of *k_deg_* indicate that protein degradation rates for many proteins are slower in *ATG5*^−/−^ and *ATG7*^−/−^ cells in comparison to wild-type cells (Figure 3). Based on the exclusion criteria described above, we quantified the change in degradation rate upon inhibition of autophagy (*Δk_deg_*) of 2,013 proteins by comparing datasets obtained from *ATG5*^−/−^ and *ATG7*^−/−^ cells with WT^+vector^ cells (Table S6). The median difference in protein degradation rates (Δ*k_deg_*) between WT^+vector^ and *ATG5*^−/−^ cells, and WT^+vector^ and *ATG7*^−/−^ cells was ∼0.03 d^−1^. Interestingly, the degradation rates of a small subset of proteins actually appeared to increase in the autophagy deficient cells, suggesting that compensatory clearance mechanism may be becoming activated in *ATG5*^−/−^ and *ATG7*^−/−^ cells (see below).

### Gene Ontology Enrichment within the Δk_deg_ Distribution

**Figure 4:**
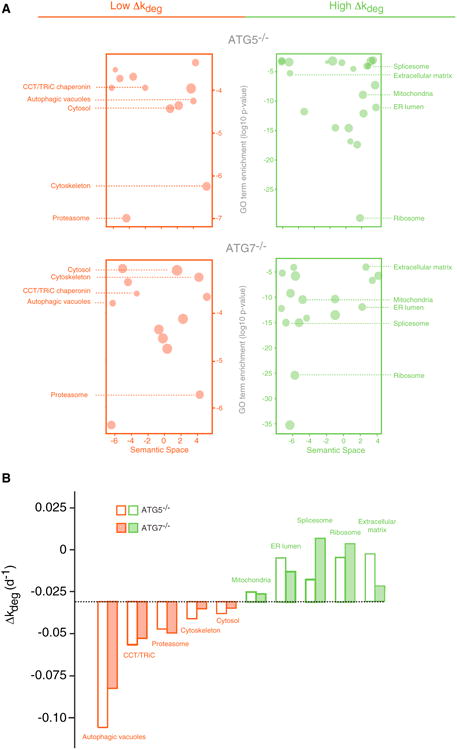
Degradation Kinetics of Proteins Belonging to Specific Cellular Component Gene Ontology Categories Are Differentially Impacted by the Inhibition of Autophagy (A) Gene Ontology (GO) term enrichments of proteins with high (green) or low (red) Δ*k_deg_* values were organized based on semantic similarity and visualized by REViGO. × axis represents the semantic similarity between GO terms based on overlap of their constituent proteins, and y axis is the Benjamini-corrected p values of statistical significance for GO category enrichment; the symbol size is correlated to the number of proteins mapped to each GO term. (B) The median of Δ*k_deg_* values for proteins mapped to GO categories indicated in (A). See also Table S7 for a complete list of GO term enrichments and genes mapped to each term.

The impact of *the ATG5*^−/−^ and *ATG7*^−/−^ mutations on degradation rates is variable between proteins, with *Δk_deg_* ranging from∼0.10 d^−1^ to +0.05 d^−1^ for most proteins (Figure 3B). We analyzed the statistical enrichment of specific Gene Ontology (GO) terms in the outer limits of this distribution. The analysis was conducted using the software tool *GOrilla* and visualized by *ReviGO* (Eden et al., 2009; Supek et al., 2011). Using this analysis, we identified GO terms with statistically significant high and low Δ*k_deg_* values relative to the entire quantified proteome in both *ATG5*^−/−^ and *ATG7*^−/−^ cells (Figure 4; Table S7). Proteins in our dataset associated with autophagosomes were significantly stabilized in autophagy deficient cells. This subset included known autophagosome interacting proteins FYCO1 (Pankiv et al., 2010) and subunits of the HOPS complex (Jiang et al., 2014) (Table S7). Cytosolic proteins, known to be the predominant substrates of autophagy, were enriched in the subset of the proteome with low Δ*k_deg_.* values, whereas proteins mapped to the mitochondria, endoplasmic reticulum, and the extracellular matrix were less impacted by the two mutations.

**Figure 5:**
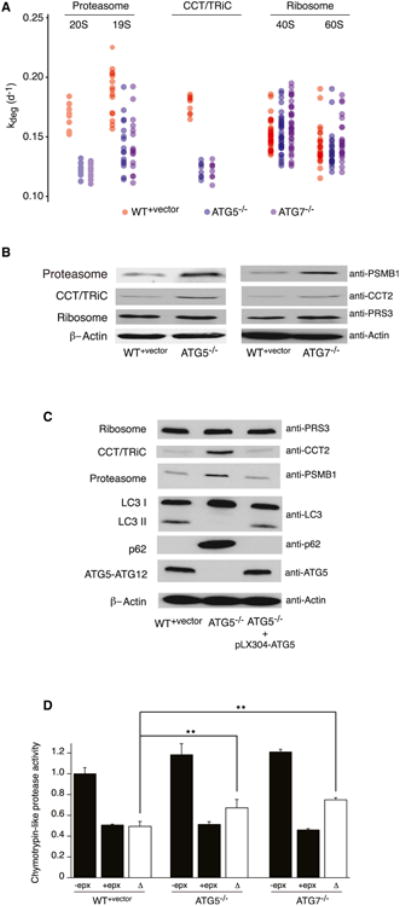
The Inhibition of Autophagy Impacts the Stability and Expression Levels of Protein Complexes Involved in Proteome Homeostasis (A) Degradation rates of subunits of the ribosome, proteasome, and CCT/TRiC in WT^+vector^, *ATG5^-^*^/^*^-^* and *ATG7^-^*^/^*^-^* cells. (B) The proteasome and CCT/TRiC accumulate in autophagy-deficient cells while ribosome levels remain unchanged. (C) Re-expression of *ATG5* in *ATG5*^−/−^ cells restores autophagy and reduces expression levels of the proteasome and CCT/TRiC. (D) Autophagy-deficient cells have higher levels of proteasome activity in cell free assays (n = 5, **p < 0.01). See also Figure S5.

Additionally, a number of GO terms related to non-autophagy degradation pathways were among the most highly enriched in the subset of proteins with low Δ*k_deg_* values. In particular, the subunits of the 19S and 20S proteasome showed dramatic decreases in degradation rates (Figure 5A). Another set of GO terms whose constituent proteins were significantly stabilized in *ATG5*^−/−^ and *ATG7*^−/−^ cells were those related to cellular protein folding and molecular chaperones. Most significantly, the CCT/TRiC chaperonin was dramatically stabilized in *ATG5*^−/−^ and *ATG7*^−/−^ cells. Conversely, the degradation rates of many proteins involved in mRNA processing and protein synthesis, including the constituents of the splicesome and ribosome, were unaffected by the mutations (Figure 5A).

Changes in the stability of the proteasome, ribosome, and CCT/TRiC chaperonin were reflected in their steady-state levels (Figure 5B). Western blot analysis indicated that proteasome and CCT/TRiC levels were significantly increased in both *ATG5*^−/−^ and *ATG7*^−/−^ cells, whereas the ribosome levels remained unaffected. mRNA transcript levels for the analyzed subunits of the proteasome, CCT/TRiC, and ribosome were not altered by the Atg5^−/−^ mutation (Figure S5), supporting the conclusion that the observed changes in expression levels were caused by altered protein stability. Re-expression of *ATG5* in *ATG5*^−/−^ cells, engineered with synonymous mutations to avoid targeting by anti-*ATG5* sgRNA (see Supplemental Experimental Procedures), was able to re-establish autophagic flux and selectively diminish the expression levels of the proteasome and CCT/TRiC (Figure 5C).

### The Activity of UPS in *ATG5*^−/−^ and *ATG7*^−/−^ Cells

The above results indicate that the deletion of *ATG5* and *ATG7* not only inhibits the autophagy pathway but also potentially impacts protein flux through the UPS due to stabilization of the proteasome. To examine the effects of *ATG5 and ATG7* deletions on the UPS, we measured the total cellular proteasome activity in cell-free assays (Figure 5D). Our results indicate that proteasome activity is increased by ∼25% in both deletion backgrounds, paralleling the increase in intracellular proteasome levels. Thus, clearance of proteins in autophagy-impaired cells is impacted by at least two mechanisms. First, the elimination of autophagosome formation may decrease the clearance rates of autophagy substrates. Second, the increase in proteasome levels could potentially increase the degradation rate of some UPS substrates, mitigating some of the impact of autophagy impairment. Thus, for an individual protein, the measured Δ*k_deg_* potentially encompasses the sum contribution of at least two opposing effects.

**Figure 6:**
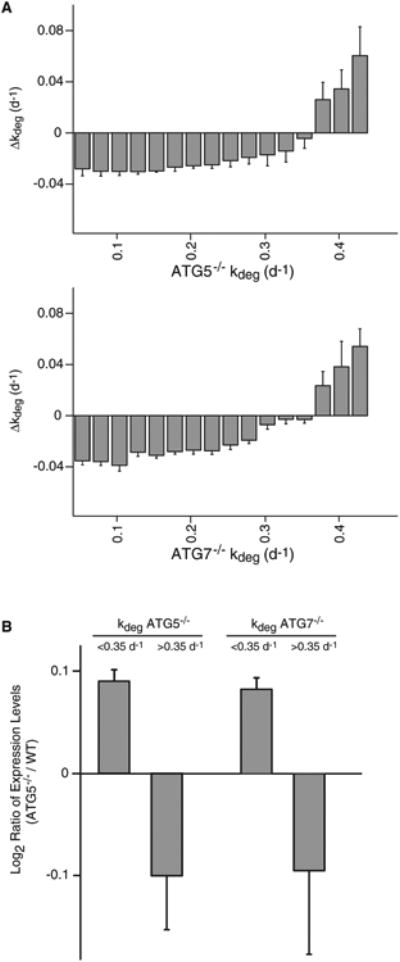
The Differential Impact of Autophagy Inhibition on Long-Lived and Short-Lived Proteins (A) The average Δ*k_deg_* for subsets of proteins with varying degradation rates in *ATG5*^−/−^ and *ATG7*^−/−^ cells. The trend is consistent with the idea that the change in degradation rates of long-lived proteins is primarily due to reduced flux through the autophagy pathway whereas short-lived proteins may be de-stabilized due to increased proteasome levels. Bars indicate the mean of Δ*k_deg_* measurements for subsets of proteins with the indicated range of *k_deg_* values, and error bars indicate SEM. (B) Steady-state SILAC analysis indicates that, as a group, relative expression levels of long-lived proteins are increased and relative expression levels of short-lived proteins are decreased in *ATG5*^−/−^ and *ATG7*^−/−^ cells. Bars indicate the mean of the log_2_ ratio of expression levels between *ATG5*^−/−^ and WT^+vector^ cells for subsets of proteins with the indicated range of *k_deg_* values, and error bars indicate SEM.

To gain more insight into the relative magnitude of each of the above effects, we examined the distribution of Δ*k_deg_* for subsets of proteins with varying degradation rates in autophagy-deficient cells (Figure 6A). If we assume that UPS is a principal route of protein clearance in *ATG5*^−/−^ and *ATG7*^−/−^ cells, we would expect that proteins with faster rates of degradation in these cells should generally have more positive Δ*k_deg_ values.* In other words, proteins that have higher rates of flux through the UPS should become more destabilized as a result of increases in proteasome levels. Indeed, for the most rapidly degrading proteins in *ATG5*^−/−^ and *ATG7*^−/−^ cells, Δ*k_deg_* is generally (though not uniformly) more positive. In fact, as a group, proteins whose rate of degradation exceeds ∼0.35 d^−1^ have a positive Δ*k_deg_* value and are less stable in autophagy-deficient cells in comparison to wild-type cells. However, for more long-lived proteins (*k_deg_ <* 0.3 d^−1^), Δ*k_deg_* values are negative and relatively constant as a function *of k_deg_ In this* subset of stable proteins, the increase in proteasome levels does not seem to greatly increase the rate of flux through the UPS. We suggest that although long-lived proteins can be degraded by the UPS (e.g., even for the most stable proteins, *k_deg_* generally decreases by less than 50% in autophagy-deficient cells), their flux through this pathway is not rate-limited by the level of the proteasome. Rather, for these stable proteins, targeting and ubiquitination may establish the rate of flux through the UPS. For this subset of stable proteins, the measured Δ*k_deg_* values are unaffected by changes in proteasome levels induced by the *ATG5*^−/−^ and *ATG7*^−/−^ mutation and may reflect true rates of basal autophagy.

To obtain additinoal support for upregulation of UPS in autophagy impaired cells, we analyzed the global impact of *ATG5* and *ATG7* mutations on steady-state protein levels by a standard SILAC experiment. WT^+vector^ cells were grown in isotopically labeled media for a number of passages in order to fully label the proteome. Unlabeled *ATG5*^−/−^ cells and labeled WT+vector cells were grown to confluency and protein extracts from quiescent cultures were combined at a 1:1 ratio and analyzed by LC-MS/MS as described in Experimental Procedures. In this experiment, ratios of labeled to unlabeled spectra report on changes in steady-state expression levels of proteins induced by impairment of autophagy. Our data indicate that, as a group, proteins that have slow rates of degradation (k_deg_ < 0.35 d^−1^) have increased relative expression levels in autophagy-impaired cells (Figure 6B; Table S8). Conversely, rapidly degrading proteins (kdeg > 0.35 d^−1^) that are likely to be enriched in proteasome substrates have generally lower relative expression levels in autophagy-impaired cells. However, it is important to note that the overall correlation between *k_deg_* and changes in expression levels is weak (Pearson correlation coefficient of 0.21 and 0.20 for *ATG5*^−/−^ and *ATG7*^−/−^, respectively), suggesting that for many proteins, *ATG* mutations can alter expression levels independent of their effect on degradation rates.

### Coordinated Degradation of Multi-subunit Protein Complexes by Autophagy

**Figure 7:**
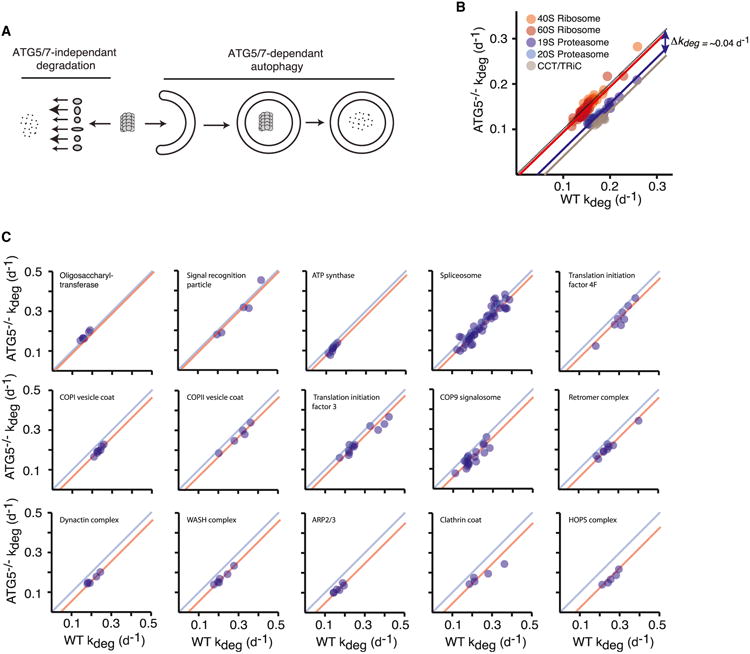
Subunits of Protein Complexes Are Degraded as a Unit by Autophagy (A) Proposed model for the degradation of protein complexes. Subunits of protein complexes may be degraded as a unit by ATG5/7-dependent autophagy or at distinct rates by ATG5/7-independent pathways. (B) Comparison of degradation rates of ribosome, proteasome, and CCT/TRiC subunits in WT^+vector^ and ATG5^−/−^ cells. The data indicate that the degradation rates of most subunits belonging to a complex are decreased by a relatively constant factor (Δ*k_deg_*). (C) This general trend is observed for a number of stable complexes. See also Figure S6.

The mechanism of autophagy involves the capture of relatively large volumes of cytoplasm within vesicles, suggesting that large stable protein complexes are captured and degraded as a single unit. This is in contrast to the proteasome, which is generally believed to degrade individual monomeric proteins (Coux et al., 1996; Kish-Trier and Hill, 2013). Thus, subunits of large protein complexes can potentially be degraded by two distinct mechanisms: one where they dissociate from the complex prior to degradation by the proteasome, and one where they are degraded in conjunction with other complex subunits by autophagy (Figure 7A). In the former mechanism, each subunit of the complex could potentially be degraded at a distinct rate, whereas in the latter mechanism, degradation rates of subunits are expected to be similar. Thus, if the measured Δ*k_deg_* values for long-lived stable protein complexes are indeed a true measure of basal autophagy, we would predict that these values would be similar for subunits of stable protein complexes.

Indeed, we observe that Δ*k_deg_* is remarkably uniform among subunits of most stable complexes. As an example, Figure 7B compares the degradation rates of the subunits of the proteasome, CCT/TRiC, and ribosome between WT^+vector^ and *ATG5*^−/−^ cells. The data indicate substantial variation between *kdeg* of complex subunits within each genetic background. However, Δ*k_deg_* values of subunits are relatively uniform within each complex. This phenomenon globally holds for most stable complexes in our dataset and is also observed in *ATG7*^−/−^ cells (Figures 7C and S6). Analysis of complexes for which we were able to quantify multiple subunits indicates that Δ*k_deg_* values within subunits of complexes are relatively uniform. However, inter-complex Δ*k_deg_* values are variable, reflecting differential rates of basal autophagy for each complex.

## Discussion

Previous proteomic analyses had sought to globally characterize the selectivity of autophagy either by analyzing changes in steady-state levels of proteins upon autophagic inhibition (Mathew et al., 2014) or identifying subsets of proteins that are co-isolated with autophagosomes (Dengjel et al., 2012; Mancias et al., 2014). Here, we instead utilized a dynamic proteomic approach to quantitatively analyze rates of degradation in autophagy-deficient cells and measured relative contributions of autophagy to proteome turnover. This strategy offers two important advantages over previous proteomic approaches. First, it can analyze the impact of autophagy impairment on protein flux independently of its potential effects on transcription and protein synthesis that may also influence steady-state expression levels. Second, it can analyze the impact of autophagy on protein flux under conditions where a small minority of the steady-state protein population is associated with autophagosomes, and is therefore particularly well suited for analysis of autophagy under basal conditions.

Our current study highlights a number of principles regarding the selectivity of autophagy and the division of labor between autophagy and ATG5/7-independent degradation pathways. Our results indicate that autophagy is highly redundant with alternative degradation pathways. A large fraction of the proteome can be degraded, to different extents, by both ATG5/7-dependent and ATG5/7-independent degradation pathways. However, the range of protein degradation rates by ATG5/7-independent pathways (reflected in the distribution of *k_deg_s* in autophagy-impaired cells) is significantly broader than the autophagy pathway (reflected in the distribution of Δ*k_deg_s* between wild-type and autophagy-impaired cells). The results suggest that whereas short-lived proteins are degraded almost exclusively by ATG5/7-independent pathways, long-lived proteins can be robustly degraded by both ATG5/7-dependent and ATG5/7-independent pathways. Even among the most stable proteins, ATG5/7-dependent autophagy typically accounts for significantly less than 50% of the basal protein flux.

Although the range of basal protein degradation rates by autophagy is narrower than ATG5/7-independent pathways, there are clear biases in autophagic target selection. For example, we show that the proteasome and the CCT/TRiC are robustly targeted for autophagy, while the ribosome is largely protected from autophagy under basal conditions. Thus, the selectivity of basal autophagy may establish a self-compensatory system of protein degradation and synthesis. Because the proteasome is an autophagic substrate under basal conditions, the inhibition of autophagy results in the stabilization of the proteasome complex and enhancement of degradative flux through the UPS. Conversely, the ribosome and other complexes involved in protein synthesis are excluded from autophagy. As a result, increases in protein synthesis are prevented under conditions where autophagic protein degradation is compromised. Additionally, the stabilization of chaperones may counteract proteostatic disruptions resulting from the inhibition of autophagy.

An increase in proteasome activity within autophagy-deficient cells is consistent with recent results indicating that proteasomes can be selectively targeted for degradation by autophagy (Dengjel et al., 2012; Marshall et al., 2015). Upon impairment of autophagy, the resulting increase in proteasome levels destabilizes some short-lived proteins in the cell but does not impact the degradation of long-lived proteins by the UPS. We suggest that unlike transient proteins, the degradation of stable proteins by the UPS may be rate limited not by proteasome levels but rather by the rate of polyubiquitination. It is important to note that previous studies had indicated that autophagy impairment results in inhibition of the degradation of specific substrates (e.g., p53) by the UPS (Korolchuk et al., 2009). These studies showed that p62 accumulates as aresult of autophagic inhibition and impedes the degradation of specific ubiquitinated proteins by the proteasome. Thus, autophagic inhibition may influence the degradation of UPS substrates by two opposing mechanisms: changes in proteasome levels and accumulation of p62.

The degradation of ribosomes and other organelles by autophagy has been documented by a number of previous studies. For example, ribosomes are typically found to be sequestered in autophagosomes when autophagy is induced by starvation (Kraft et al., 2008). The selective autophagic degradation of organelles is mediated by specialized receptors (e.g., Nix or p62 for autophagy) or requires other modifying enzymes (e.g., Ubp3-Bre5 deubiquitinating enzyme complex or ribophagy). Interestingly, we have shown here that the basal turnover kinetics of ribosomes and organelles such as mitochondria are not strongly impacted by the impairment of autophagy in quiescent cells. We speculate that starvation ordamaged-induced selective degradation of organelles requires specialized receptors or factors to override their natural tendency to be excluded from basal autophagy. For example, in the case of ribophagy, it is known that the Ubp3-Bre5 deubiquitinating enzyme complex is required for the selective degradation of the 60S ribosome in yeast and that Ltn1/Rkr1, a ribosome-associate E3 ligase, inhibits this degradation by ubiquitinating the ribosomal subunit Rpl25 (Ossareh-Nazari et al., 2014). Thus, under basal conditions, ribosomes may harbor post-translational modifications that protect them from autophagy, and these modifications may need to be removed to induce their selective degradation by autophagy under starvation.

In recent years, there has been a general shift from the perception of autophagy as a non-selective starvation response to a selective degradation mechanism capable of targeting specific proteins and organelles for clearance. Here, we have provided a large-scale census of the contribution of basal autophagy to constitutive turnover of proteins in quiescent human fibroblasts. Our results demonstrate that the contribution of autophagy to constitutive protein turnover is variable within the proteome, and that this selectivity results in the stabilization of specific protein complexes responsible for maintaining proteome homeostasis under conditions where autophagy is inhibited. The observed selectivity of basal autophagy may reflect the presence of a compendium of cargo-specific autophagic receptors within the cell or a range of affinities toward a more limited set of receptors. Whether the magnitude and selectivity of autophagic flux observed in our studies is specific to quiescent fibroblasts or can be extended to other cell types remains to be determined. The proteomic methodology outlined in this study may provide a useful global approach for elucidating the mechanisms of autophagic selectivity in diverse cell types and environmental conditions.

## Experimental Procedures

### Generation of *ATG5*^−/−^ *and ATG7*^−/−^ Fibroblasts

*ATG5*^−/−^ and *ATG7*^−/−^ human primary fibroblasts were generated by utilizing the LentiCRISPR v1 (Addgene plasmid 49535) vector and LentiCRISPR v2 (Addgene plasmid 52961) co-expressing the mammalian codon-optimized Cas9 nuclease and sgRNAs (Sanjana et al., 2014; Shalem et al., 2014). The puromycin selection marker in LentiCRISPR v2 sgRNAs was substituted by blasticidin. sgRNAs were designed using the CRISPR design tool (http://crispr.mit.edu) to minimize potential off-target effects. Five sgRNAs targeting three different exons in each gene were designed. The sequences of sgRNAs that ultimately produced successful deletion clones and were further analyzed are listed in Table S1. HEK293FT cells were seeded in six-well plates 1 day prior to transfection at a density of 500,000 cells per well. HEK293FT cells were transfected with LentiCRISPRv1, pMG2.G (Addgene), and psPAX2 (Addgene) plasmids in DMEM (10569-010) using Lipofectamine 3000 (Life Technologies) following the recommended protocol. At 72 hr post-transfection, the cell culture media was collected and filtered using Steriflip-HV sterile centrifuge tube top filter (Millipore). HCA2-hTertcellswere plated in 80% confluency, and 2 ml resulting lentivirus was used for infection. 72 hr post-infection, cells were cloned by limiting dilution and individual clones were isolated. 72 hr post-infection, cells were selected using 4 ng/ml blasticidin for 5 days and used for further analysis. Subsequently, cells were cloned by limiting dilution and individual clones were isolated.

To re-express *ATG5 in ATG5*^−/−^ cells, *H. sapiens ATG5* autophagy-related 5 homolog in pLX304 lentiviral plasmid (clone HsCD00418067) was obtained from Harvard PlasmID database. Gibson cloning was used to introduce silent mutations in the target region of sgRNAto prevent deletion by Cas9 (Supplemental Experimental Procedures). The cloned vectors were used for viral production, and Atg5^−/−^ cells were infected with the resulting lentiviruses as described above. At 72 hr post-infection, cells were selected using 4 μg/ml blasticidin for 5 days and used for further analysis.

### Stable Isotope Labeling

The media utilized for isotopic labeling was Eagle's minimum essential medium (ATCC) supplemented with 15% dialyzed fetal bovine serum (Thermo Scientific), 100 U/ml penicillin, and 100 U/ml streptomycin. Cells were gradually adapted to this media using the procedure outlined in Table S2. Cells were then plated at a density of 500,000 cells per 10 cm plate. 8 days after plating, the confluent quiescent cultures were switched to MEM labeling media for SILAC (Thermo Scientific) supplemented with L-arginine:HCl (^13^C6, 99%) and L-lysine:2HCl (^13^C6, 99%; Cambridge Isotope Laboratories) at concentrations of 0.1264 g/l and 0.087 g/l and 15% dialyzed fetal bovine serum (Thermo Scientific). Cells were collected after 0, 2, 4, and 6 days of labeling, washed with PBS and cell pellets were frozen prior to further analysis. In order to assess the precision of our measurements, biological replicate experiments were conducted for WT^+vector^ and *ATG5*^−/−^ experiments and cells were collected after 4 days for analysis.

In order to measure changes in steady-state protein levels by standard SILAC, WT^+vector^ and *ATG5*^−/−^ cells were gradually adapted to Eagle's minimum essential medium (ATCC) supplemented with 15% dialyzed fetal bovine serum (Thermo Scientific), 100 U/ml penicillin, and 100 U/ml streptomycin. Then, WT^+vector^ cells were passaged in MEM labeling media for SILAC (Thermo Scientific) supplemented with L-arginine:HCl (^13^ C6, 99%) and L-lysi-ne:2HCl (^13^ C6, 99%; Cambridge Isotope Laboratories) at concentrations of 0.1264 g/l and 0.087 g/l and 15% dialyzed fetal bovine serum (Thermo scientific) for eight passages. Cells were then plated at a density of 500,000 cells per 10-cm plate. 8 days after plating, confluent quiescent cells were collected and washed with PBS, and cell pellets were frozen prior to further analysis. Following extraction (see below), equal protein amounts of WT^+vector^ and *ATG5*^−/−^ were mixed before mass spectrometric analysis.

### Mass Spectrometry Sample Preparation and Protein Digestion

A buffer containing 50 mM Tris-formic acid (FA) (pH = 7.8), 150 mM sodium chloride, 1% sodium deoxycholate, 2% IGEPAL CA-630, 2% SDS, and protease/phosphatase inhibitor cocktail tablets (Roche Applied Science) was used for cell lysis. 200 μl lysis buffer was added to ∼10^6^ cells. The samples were sonicated using a high-energy sonicator (Qsonica) with 5-s bursts for 30 s (non-continuous) on ice. The samples were then centrifuged for 30 min at 4°C at 20,000 × *g.* The supernatant were transferred to a new Eppendorf tube, while the cell debris pellet at the bottom and the lipid layer on the top of the supernatant were avoided. Protein concentration were measured by the bicinchoninic acid assay (BCA) kit (Pierce Biotechnology), and the final protein concentration was adjusted to 1 μg/μl. Reduction of protein disulfide bonds was performed with 3 mM Tris (2-carboxyethyl) phosphine (TCEP) at 37°Cfor30 min in an Eppendorf Thermomixer (Eppendorf), and protein alkylation was performed with 20 mM iodoacetamide at 37°C for 30 min in darkness. A SOD procedure was employed to derive tryptic peptides for LC-MS/MS analysis. Proteins were first precipitated by stepwise addition of nine volumes of chilled acetone and overnight incubation under —20°C to remove undesirable components in the sample matrix, e.g., detergents, non-protein cellular components. After centrifugation at 4°C and 20,000 × *g* for 30 min, the supernatant was discarded and the protein pellet was washed with 800 μl chilled acetone/water mixture (85/15, v/v %), followed by solvent removal and air drying (∼5 min). The protein pellet was then re-suspended in 80 μl Tris-FA buffer, and a two-step digestion procedure was performed as previously described (An et al., 2015; Duan et al., 2009). In the first step, trypsin at an enzyme:substrate ratio of 1:40 (w/w) was added to the solution and the mixture was incubated at 37°C for 6 hr with constant vortexing to cleave the proteins into large peptide fragments. In the second step, a same amount of trypsin was added to reach a final enzyme: substrate ratio of 1:20 (w/w), and the mixture was incubated at 37°C overnight (12 hr) with constant vortexing to achieve complete cleavage of proteins. Digestion was terminated by adding 1% FA to the solution, followed by centrifugation at 20,000 × *g* for 30 min at 4°C to remove impurity particles. The supernatant containing tryptic peptides derived from 4 μg proteins from each sample was used for LC-MS/MS analysis.

In order to increase proteome coverage, high-pH fractionation was conducted for some time points in the experiment (all time points for *ATG7*^−/−^ and 4-day time points for WT^+vector^ and *ATG5*^−/−^) using the Pierce High pH Reversed-Phase Peptide Fractionation Kit (catalog #84868). Eight different elution buffers were made in 0.1% trimethylamine, with 5%, 7.5%, 10%, 12.5%, 15%, 17.5%, 20%, and 50% acetonitrile added. After conditioning the column with acetonitrile and 0.1% TFA, the samples are added and then centrifuged. A water wash was conducted to wash away any residual salts before the eight elutions were collected in fresh tubes. Samples were dried down and subsequently re-suspended in 50 μl 0.1% formic acid.

### Nano-LC-MS/MS

Tryptic peptides were analyzed using an Eksigent (Δublin) ekspert nano-LC 425 system coupled to an Orbitrap Fusion Tribrid mass spectrometer (Thermo Scientific). The mobile phases consisted of 0.1% FA in 2% acetonitrile (ACN) for A and 0.1% FA in 88% ACN for B. Samples were loaded onto a reversed-phase trap (300 mm inner diameter [ID] × 0.5 cm, packed with Zorbax5-mm C18 material), with 1*%* mobile phase B at a flow rate of 10 μL/min, and the trap was washed for 3 min. A series of nanoflow gradients at a flow rate of 250 μl/min was used to back-flush the trapped samples onto the nano-LC column (75 mm ID × 100 cm, packed with 3-μm particles) for chromatographic separation. The nano-LC column was heated at 52°C so that both chromatographic resolution and reproducibility could be significantly improved. A 3 hr shallow gradient was used to achieve sufficient peptide separation. The gradient profile was listed as follows: 3% B for 3 min, 3%–6% B for 5 min, 6%–28% B for 125min,28%–50% B for 10 min,50%–97% Bfor1 min, and isocraticat97% B for 18 min, and then the column was equilibrated with 3% B for 18 min.

An Orbitrap Fusion Tribrid mass spectrometer was used for ion detection. The data-dependent product ion mode was selected for all analyses. One scan cycle consisted of one MS1 survey scan (m/z 400–1,500) at a resolution of 120,000 and 20 MS2 scans to fragment the 20 most abundant precursor ions in the survey scan via high-energy collision dissociation (HCD) activation. For MS1 survey scans, automatic gain control (AGC) target was set to 4 × 10^5^, and peptide precursors with charge states of 2–7 were sampled for MS2. Dynamic exclusion was enabled with the following settings: repeat count = 1, exclusion duration = 60s, and mass tolerance = ±10 ppm. Monoisotopic precursor selection was turned on. The instrument was run in top speed mode with a cycle time of 3 s. MS2 scans was performed with ddMS2 Orbitrap (OT)-HCD, and the isolation window was set at 1 Th with quadrupole. The normalized collision energy was 35%, and tandem mass spectra were analyzed with a resolution of 15,000. The MS2 AGC target was set to 5 × 10^4^, and the maximal injection time was 50 ms with centroid data type of one microscan.

### Quantitative Proteomic Data Analysis

Peptides were identified from MS/MS spectra by searching against the *H. sapiens* Swiss-Prot database using the integrated Andromeda search engine with the software MaxQuant (Cox et al., 2011). SILAC peptide and protein quantification was performed with MaxQuant using the parameter settings listed in Table S3. For each peptide, the heavy/light (H/L) ratio was determined by a regression model fitted to all isotopic peaks within all scans that the peptide eluted in. H/L ratio for each protein was determined as the median of all the peptides assigned to the protein (de Godoy et al., 2008). For a given protein, H/(H+L) ratio was calculated based on the H/L ratio from MaxQuant outputs. All H/(H+L) ratios at all time-points were combined to obtain an aggregated plot for the kinetics of labeling. The aggregated plots were fitted to a single exponential function by least-square fitting to obtain the first-order degradation rate constant (*k_deg_*) for each protein.

## Supplementary Material



## Highlights

- Contribution of autophagy to protein turnover was quantified by dynamic proteomics
- Autophagy contributes to the basal turnover of much of the proteome at varying levels
- Subunits of protein complexes are degraded as a unit by autophagy
- Proteasome and CCT/TRiC are targeted by basal autophagy, but the ribosome is protected
