# Supplementary material for: Global Analysis of Cellular Protein Flux Quantifies the Selectivity of Basal Autophagy
Source: Cell Rep. Author manuscript; Available in PMC 2017 Jun 14. (PMC5470642; doi:10.1016/j.celrep.2016.02.040)
Supplement: Supplementary file 1 [file NIHMS867311-supplement-supplement_1.pdf]

Cell Reports, Volume *14*

Supplemental Information

Global Analysis of Cellular Protein Flux Quantifies the Selectivity of Basal Autophagy

Tian Zhang, Shichen Shen, Jun Qu, and Sina Ghaemmaghami

Supplemental Experimental Procedures

Accession Numbers

The MS-based proteomics data and detailed protocols have been deposited at the ProteomeXchange Consortium (http://proteomecentral.proteomexchange.org) via the PRIDE partner repository. The data have been divided into three parts:

1. The dynamic time-resolved isotopic labeling experiments (data set identifier PXD003562)

2. The SILAC experiments for analyzing changes in steady-state expression levels (data set identifier PXD003558)

3. ^{15}N labeling experiments for conducting isotopomer analyses (data set identifier PXD003559**)**

Cell culture

Human primary fibroblasts were maintained in Eagle's Minimum Essential Medium (ATCC) supplemented with 15% fetal bovine serum (Invitrogen), 100U/mL penicillin, 100U/mL streptomycin at 37°C with 5% CO_2. Human embryonic kidney (HEK) cell line 293FT (Life technologies) was maintained in Dulbecco's modified Eagle's medium (Life technologies) supplemented with 10% fetal bovine serum (Invitrogen), 100U/mL penicillin, 100U/mL streptomycin at 37°C with 5% CO_2.

SURVEYOR assay

Genomic DNA was extracted from cells using the Quick Extract DNA extraction solution (Epicentre) following the recommended protocol. Briefly, cells were resuspended in QuickExtract solution and incubated at 65°C for 15 minutes and 98°C for 10min. Approximately 600 bp and 700 bp regions surrounding the CRISPR target site for Atg5 and Atg7 was PCR amplified (see Table S1 for primer sequences), and products were purified using QiaQuick Spin Columns (Qiagen) following the manufacturer's protocol. A total of 400 ng of the purified PCR products were mixed with 2 μl 10X Taq polymerase PCR buffer (Life technologies) and ultrapure water to a final volume of 20 μl, and subjected to a re-annealing process to enable hetero-duplex formation: 95°C for 10min, 95°C to 85°C ramping at – 2°C/s, 85°C to 25°C at – 0.25°C/s, and 25°C holding for 1 minute. After re-annealing, products were treated with SURVEYOR nuclease and SURVEYOR enhancer S (Transgenomics) following the manufacturer's recommended protocol. Resulting fragments were analyzed on 10%

polyacrylamide gels, stained with SYBR Gold DNA stain (Life Technologies) for 30 minutes and imaged with a Gel Doc gel imaging system (Biorad).

Cell cycle analysis by flow cytometry

Wildtype and $Atg5^{-/-}$ were cultured as described above and Cells were collected and 1 x 10^6 cells were centrifuged at 300 x g for 5 minutes and washed once with 1 x PBS. While mixing and resuspending cells, 1 mL of ice cold 70% ethanol was slowly added to fix cells. 200uL of fixed cells were transferred to new tube and centrifuged at 300 x g for 5 minutes and wash once with PBS. 200 µL of Muse Cell Cycle reagent from Muse Cell Cycle Kit (Millipore) was used for each tube. Cells were incubated for 30 minutes at room temperature in the dark. Muse Cell Analyzer (Millipore) was used to analyze the DNA content of each sample.

Measurement of viability after amino acid starvation

$WT^{+vector}$, $Atg5^{-/-}$ and $Atg7^{-/-}$ clones were seeded into 6-well plates one day prior to amino acid starvation at a density of 200,000 cells per well. Cells were washed with PBS and 2mL Earle's Balanced Salt Solution was added. After 24, 36, 48 hours treatment, all cells were collected and 200 µL of Muse Count & Viability regent from Muse Count & Viability Assay Kit (Millipore) was added to each tube. After 30 minutes incubation at room temperature, Muse Cell Analyzer (Millipore) was used to analyze samples. For each sample, three biological replicates were performed and averaged.

Proteasome activity assay

Proteasome activity was measured by using 20S Proteasome activity assay kit (Millipore). 75 µg of total protein in 50 µL cell lysate was incubated in assay buffer containing 25 nM HEPES, pH 7.4, 0.05 mL EDTA, 0.05% mM Nondiet P-40, 0.001% SDS and 25ug of the proteasome substrate, LLVY-AMC, for 90min at 37°C. Enzyme activity was measured by monitoring release of the fluorescent LLVY-AMC cleavage product, 7-amino-4-methylcoumarin. Activity measurements from parallel reactions containing 10 μM of the proteasome inhibitor epoxomicin were subtracted from the activity measurements obtained without the inhibitor.

Western blotting

Cells were lysed with ice-cold lysis buffer (10mM pH8.0 Tris-HCl, 0.15M NaCl, 0.5% NP-40, 0.48% SDS). Cell lysates were centrifuged at 13,000 rpm for 10 min. For Western blot analysis, 30 μg was separated by electrophoresis in 10% polyacrylamide gels and transferred to polyvinylidene difluoride (PVDF) membranes using Trans-Blot SD Semi-Dry Electrophoretic Transfer Cell (Biorad). After 1h incubation at room temperature in TBST/5% nonfat milk, the membrane was incubated with the indicated antibodies at 4°C overnight. The membranes were then washed with TBST/0.1% Tween 20,

and the corresponding secondary antibodies were applied to the membranes for 1h at room temperature. The membranes were then washed with TBST/0.1% Tween 20, and the detection of signal was done with an enhanced chemiluminescence detection kit (Pierce). The primary antibodies and the corresponding dilutions utilized for the Western blots were: Anti-RPS3 Antibody: 1:1000 (Millipore), CCT2 Antibody: 1:1000(Cell Signaling Technologies), 20S Proteasome α2 Antibody (B-4): 1:200 (Santa Cruz Biotechnology), p27 antibody (F-8): 1:200 (Santa Cruz Biotechnology), Anti-beta Actin Antibody: 1: 2000(Abcam), Anti-p62 (SQSTM1) Antibody: 1: 1000 (MBL International), Anti-LC3 Antibody: 1:1000 (MBL International), Anti-Atg5 C-terminal Antibody: 1:1000 (Sigma Aldrich), Anti-Atg7 (D12B11) Rabbit mAb: 1:1000 (Cell Signaling Technologies).

RNA extraction and quantitative PCR

Cells were harvested and total cell RNA was extracted with TRI reagent (Sigma) following manufacturer's protocol. Extracted total RNA was quantified using Epoch Microplate Spectrophotometer (Epoch) and normalized to the same concentration. 100ng RNA was reverse transcribed with oligo-dT primers in 10uL system and proceeded to real-time PCR as templates with gene-specific primers in the presence of FastStart Universal SYBR Green Master (Roche). Pairs of primers for each gene were designed using Real Time PCR Primer Design Tool from GenScript. Quantitative real-time PR-PCR was performed twice in duplicate and triplicate reactions using a CFX Connect ™ Real-Time PCR Detection System (Biorad). Data were generated using a ΔΔCt method by normalizing the expression of the target genes to β-actin, and the values were reported as fold changes of the expression in $Atg5^{-/-}$, $Atg7^{-/-}$ cells compared to wildtype cells. Primer sequences that were utilized were as follows: β-Actin forward: CACCTCAGAAGCTGTTTCGTCCTG, reverse: CGATCCACACGGAGTACTTG; CCT2 forward: AAACCAGGACGAAACAGCTTCTGA, reverse: CAGAAGAACCTGTCGCTTCA; PSMB1 forward: ATGCAGAATGTGGAGCATGT, reverse: TGCCCTCTTTGGTCACTATG.

Stable isotope labeling for fully ^{15}N labeled samples

Cells after adaption procedure were plated at a density of 500,000 cells per 10 cm plate. 8 days after plating, the confluent quiescent cultures were switched to ^{15}N labeling medium. Cells were collected after 1d, 2d, 4d, 7d of labeling, washed with PBS and cell pellets were frozen prior to further analysis (Zhang et al., 2014). ^{15}N labeling medium was made from "Cell free" Amino Acid Mix (20AA, U-15N, 96-98%)(Cambridge Isotope Laboratories) and supplemented with 15% dialyzed fetal bovine serum (Thermo Scientific), 100U/mL penicillin, 100U/mL streptomycin. 1g is used for making 702mL labeling medium. The final amino acids concentrations in ^{15}N labeling medium are as following: Alanine: 0.1140 g/L, Arginine: 0.0499 g/L, Asparagine: 0.0940 g/L, Aspartic acid: 0.1353 g/L, Cystine: 0.01140

g/L, Glutamic acid: 0.1852 g/L, Glutamine: 0.0869 g/L, Glycine: 0.0698 g/L, Histidine: 0.0157 g/L, Isoleucine: 0.0698 g/L, Leucine: 0.1211 g/L, Lysine: 0.0527 g/L, Methionine: 0.0228 g/L, Phenylalanine: 0.0541 g/L, Proline: 0.0299 g/L, Serine: 0.0541 g/L, Threonine: 0.0741 g/L, Tryptophan: 0.0456 g/L, Tyrosine: 0.0613g/L, Valine: 0.0798 g/L.

Re-expression of ATG5 in ATG5$^{-/-}$ cells

To re-express ATG5 in *ATG5*$^{-/-}$ cells, *homo sapiens* ATG5 autophagy related 5 homolog in pLX304 lentiviral plasmid (Clone:HsCD00418067) was obtained from Harvard PlasmID Database. Gibson cloning was used to introduce silent mutations in the target region of sgRNA to knockout by Cas9. The engineered coding sequence of Atg5 is as follows:

ATGACAGATGACAAAGATGTGCTTCGAGATGTGTGGTTTGGACGAATTCCAACTTGTTTCACGCTATATC
AGGATGAGATAACTGAAAGGGAAGCAGAACCATACTATTTGCTTTTGCCAAGAGTAAGTTATTTGACGTT
GGTAACTGACAAAGTGAAAAAGCACTTTCAGAAGGTTATGAGACAAGAAGACATTAGTGAGATATGGTTT
GAATATGAAGGCACACCACTGAAATGGCATTATCCAATTGGTTTGCTATTTGATCTTCTTGCATCAAGTT
CAGCTCTTCCTTGGAACATCACAGTACATTTTAAGAGTTTTCCAGAAAAAGACCTTCTGCACTGTCCATC
TAAGGATGCAATTGAAGCTCATTTTATGTCATGTATGAAAGAAGCTGATGCTTTAAAACATAAAAGTCAA
GTAATCAATGAAATGCAGAAAAAAGATCACAAGCAACTCTGGATGGGATTGCAAAATGACAGATTTGACC
AGTTTTGGGCCATCAATCGGAAACTCATGGAATATCCTGCAGAAGAAAATGGATTTCGTTATATCCCCTT
TAGAATATATCAGACAACGACTGAAAGACCTTTCATCCAGAAACTCTTCAGGCCAGTCGCTGCAGATGGA
CAGTTGCACACACTAGGAGATCTCCTCAAAGAAGTTTGTCCTTCTGCTATTGATCCTGAAGATGGGGAAA
AAAAGAATCAAGTGATGATTCATGGAATTGAGCCAATGTTGGAAACACCTCTGCAGTGGCTGAGTGAACA
TCTGAGCTACCCGGATAATTTTCTTCATATTAGTATCATCCCACAGCCAACAGATTGA

Kinetic model

The kinetic model considered in this manuscript is based on the following assumptions:

1. Protein synthesis is a zero order process with respect to protein concentration.

2. Protein degradation occurs at a constant fractional rate that is uniform for the entire protein pool. Thus, protein degradation can be modeled as a first order process with respect to protein concentration.

3. The total protein concentration of each cell does not change during the experimental time-course (i.e. the system is at steady-state).

The first two assumptions have been shown to be general properties of cellular protein degradation (Claydon and Beynon, 2012), and the last assumption was verified experimentally (Figure S4A).

Thus, we can devise the following rate equation:

$$\frac{d[Protein]}{dt} = k_{syn} - k_{deg}[Protein] - k_{dil}[Protein] \quad (1)$$

Where k_{syn} is the first order rate constant for protein synthesis, k_{deg} is the first order rate constant for protein degradation and k_{dil} is the rate constant for dilution due to cell division. With the boundary condition $[Protein](0) = 0$, we solve for $[Protein](t)$:

$$[Protein](t) = \frac{k_{syn}}{k_{dil}+k_{deg}} - \frac{k_{syn}}{k_{dil}+k_{deg}} e^{-(k_{dil}+k_{deg})*t} \quad (2)$$

Given that the protein concentration at steady-state ($\frac{d[Protein]}{dt} = 0$) :

$$[Protein_{steady-state}] = \frac{k_{syn}}{k_{dil}+k_{deg}} \quad (3)$$

Since our protein labeling measurements (H/(H+L)) are fractional (i.e. normalized with respect to the total steady-state protein level), the observed fractional labeling is derived as:

$$Fraction\ Labeled\ Protein\ (t) = [Protein](t)/[Protein_{steady-state}] = 1 - e^{-(k_{dil}+k_{deg})*t} \quad (4)$$

Thus, the observed first order rate constant for fractional labeling ($k_{labeling}$) is derived as:

$$k_{labeling} = k_{dil} + k_{deg} \quad (5)$$

Since cells are in a quiescent state, the rate of division is zero (k_{dil}=0), and:

$$k_{labeling} = k_{deg} \quad (6)$$

Thus, the first order rate constant for degradation can be equated to the fractional rate of labeling, and used to measure the half-life of the protein:

$$halflife = ln2/k_{deg} \quad (7)$$

Table S1: Sequences for Oligonucleotides Used as Guide Sequences for CRISPR and PCR Primers for the SURVEYOR Assay, Related to Figure 1.

	**ATG5$^{-/-}$**	**ATG7$^{-/-}$**
**sgDNA_forward**	CACCTCAGAAGCTGTTTCGTCCTG	CACCGCCCGTTGCTGCCCAGCTAT
**sgDNA_reverse**	AAACCAGGACGAAACAGCTTCTGA	AAACATAGCTGGGCAGCAACGGGC
**PCR_forward**	TAGAATGTCCCACCCAACTT	GGACAGTAGAACAGCATCCTG
**PCR_reverse**	ACCAGAGTACCAGAGATATACTAAG	GTCTGGCTGCTTCAAAATGC

Table S2: Steps for Sequential Adaptation of HCA2-hTert Prior to Labeling, Related to Figure 2.

**Passage1**	25% dialyzed FBS-supplemented media: 75% Complete media
**Passage2**	50% dialyzed FBS-supplemented media: 50% Complete media
**Passage3**	75% dialyzed FBS-supplemented media: 25% Complete media
**Passage4**	100% dialyzed FBS-supplemented Media

Table S3. MaxQuant Parameter, Related to Figure 2.

Parameter	Value
Fixed modifications	Carbamidomethyl (C)
Decoy mode	revert
Special AAs	KR
Include contaminants	TRUE
MS/MS tol. (FTMS)	20 ppm
Top MS/MS peaks per 100 Da. (FTMS)	12
MS/MS deisotoping (FTMS)	TRUE
MS/MS tol. (ITMS)	0.5 Da
Top MS/MS peaks per 100 Da. (ITMS)	8
MS/MS deisotoping (ITMS)	FALSE
MS/MS tol. (TOF)	40ppm
Top MS/MS peaks per 100 Da. (TOF)	10
MS/MS deisotoping (TOF)	TRUE
MS/MS tol. (Unknown)	0.5 Da
Top MS/MS peaks per 100 Da. (Unknown)	8
MS/MS deisotoping (Unknown)	FALSE
PSM FDR	0.01
Protein FDR	0.01
Site FDR	0.01
Use Normalized Ratios For Occupancy	TRUE
Min. peptide Length	7
Min. score for unmodified peptides	0
Min. score for modified peptides	40
Min. delta score for unmodified peptides	0
Min. delta score for modified peptides	6
Min. unique peptides	0
Min. razor peptides	1
Min. peptides	1
Use only unmodified peptides and	TRUE
Modifications included in protein quantification	Acetyl (Protein N-term);Oxidation (M)
Peptides used for protein quantification	Razor
Discard unmodified counterpart peptids	TRUE
Min. ratio count	2
Re-quantify	FALSE
Use delta score	FALSE
iBAQ	FALSE
iBAQ log fit	FALSE
Match between runs	FALSE
Find dependent peptides	FALSE
Fasta file	G:\HUMAN.fasta
Labeled amino acid filtering	FALSE
Site tables	Oxidation (M)Sites.txt
Decoy mode	revert
Special AAs	KR
Include contaminants	TRUE
RT shift	FALSE
Advanced ratios	TRUE
AIF correlation	0.47
First pass AIF correlation	0.8
AIF topx	20
AIF min mass	0
AIF SIL weight	4
AIF ISO weight	2
AIF iterative	TRUE
AIF threshold FDR	0.01

Note: Tables S4-S8 are provided as separate files.

Supplemental Figure Legends

Figure S1. Validation of autophagy-deficient human fibroblasts, Related to Figure 1. (A) Genomic sequences of *ATG5*$^{-/-}$ and *ATG7*$^{-/-}$ mutant clones. Sanger sequencing chromatograms showing indel mutations in *ATG5* and *ATG7* genes targeted by CRISPR. In each figure, the top sequence (black) indicates the wildtype sequence of the targeted region and the locations of the protospacers and protospacer adjacent motifs (PAM). The colored sequences indicate the sequence of the mutated clones. The presence of overlapping sequences that deviate from wildtype is indicative of two different indel mutations in the two copies of the gene at approximately the location indicated by the red arrow.

Figure S2. During the course of isotopic labeling, fibroblasts are in a quiescent state, related to Figure 2. (A) Cell cycle analysis indicates that cells are in a quiescent state prior to the initiation of isotopic labeling. When cells become confluent (8 days after plating), over 90% of cells are in G0/G1 phase in both WT and *ATG5*$^{-/-}$ cells. (B) Increased p27 levels indicate that cells were in a quiescent state during the course of labeling.

Figure S3. Global analysis of variance in degradation rates within peptides mapped to the same protein, Related to Figure 2. The box shows the median and interquartile range (IQR) of the coefficient of variations (CV) of degradation rates for peptides encompassing a single protein. The error bar represents the entire range of CVs excluding outliers (1.5 IQR). The red lines show the CV among all peptide degradation rates within the proteome.

Figure S4. Protein levels are at a steady-state during the course of isotopic labeling and the internal amino acid pool becomes rapidly labeled upon the initiation of the labeling time-course, Related to Figure 2. (A) Total cellular protein levels remained unchanged after the initiation of isotopic labeling in quiescent cells. Protein content was determined by BCA assay and normalized by cell number. (B) The labeling kinetics of the amino acid precursor pool by istopomer analysis. Fully ^{15}N labeled media (all labeled amino acids) was added to the culture and cells were collected after 1, 2, 4 and 7 days. The mass shift of the isotopically labeled fraction (red) for a given peptide is indicative of the fraction of

amino acids that were labeled at the time of its synthesis. The predicted mass distribution for the fully labeled peptide is shown in green. For the example peptide shown (Profilin-1), it can be seen that the amino acid pool was >90% labeled after 1 day and stayed at approximately at that level for the remainder of the time-course. A similar trend was observed for the 100 most abundant peptides in our proteomic data (scatter plot). The data indicate that the amino acid pool within the cell becomes rapidly labeled upon the introduction of the labeled media to quiescent cells. Thus, the extent of fractional labeling is primarily influenced by the kinetics of protein turnover rather than amino acid uptake and recycling within the cell.

Figure S5. mRNA Levels of CCT/TRiC and proteasome subunits (CCT2 and PSMB1, respectively) are unaltered in $WT^{+vector}$ and $ATG5^{-/-}$ Cells, Related to Figure 5.

Figure S6. Subunits of protein complexes are degraded as a unit by autophagy as determined by analysis of $Atg7^{-/-}$ cells, Related to Figure 7. **(**A) Comparison of degradation rates of ribosome, proteasome and CCT/TRiC subunits in $WT^{+vector}$ and $ATG7^{-/-}$ cells. The data indicate that the degradation rates of most subunits belonging to a complex are decreased by a relatively constant factor (Δk_{deg}). This general trend is observed for a number of stable complexes (B).

Supplemental Table Legends

Table S4. Peptide Level Proteomic Data (peptide.xls), Related to Figure 2.

Table S5. Protein Level Proteomic Data (protein.xls), Related to Figure 2.

Table S6. Protein Level Kinetic Data for Determination of k_{deg}s (kdeg.xls), Related to Figure 2.

Table S7. Results of Gene Ontology (GO) Enrichment Analysis (GO.xls), Related to Figure 2.

Table S8. Protein level data for analysis of changes in steady-state expression levels (steadystate.xls), Related to Figure 6B.

(Note: More detailed information about Table S4-S8 is contained within the file)

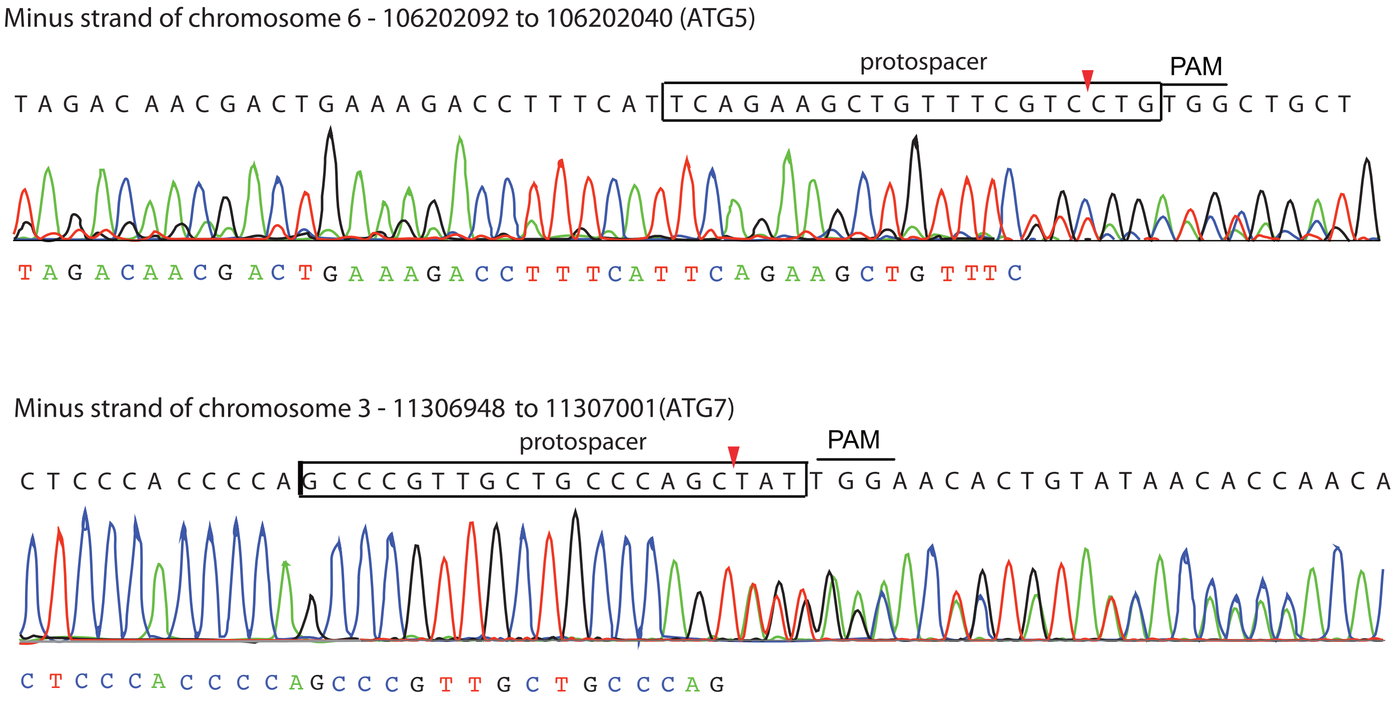
Minus strand of chromosome 6 - 106202092 to 106202040 (ATG5)
protospacer
PAM
TAGACAACGACTGAAAGACCTTTCATTCAGAAGCTGTTTCGTCCTGTGGCTGCT
TAGACAACGACTGAAAGACCTTTCATTCAGAAGCTGTTTC
Minus strand of chromosome 3 - 11306948 to 11307001(ATG7)
protospacer
PAM
CTCCCACCCCAGCCCGTTGCTGCCCAGCTATTGGAACACTGTATAACACCAACA
CTCCCACCCCAGCCCGTTGCTGCCCAG

Figure S1

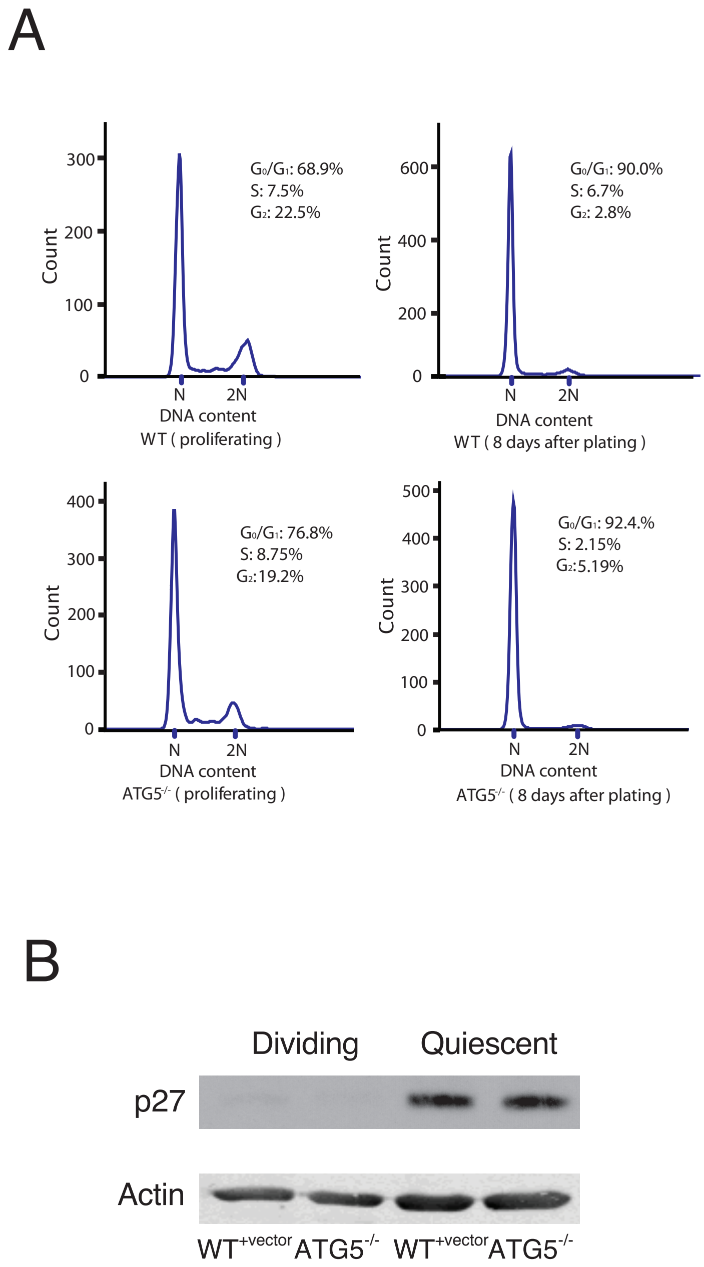
A
G0/G1: 68.9%
S: 7.5%
G2: 22.5%
Count
DNA content
WT (proliferating)
G0/G1: 90.0%
S: 6.7%
G2: 2.8%
WT (8 days after plating)
G0/G1: 76.8%
S: 8.75%
G2:19.2%
ATG5-/- (proliferating)
G0/G1: 92.4.%
S: 2.15%
G2:5.19%
ATG5-/- (8 days after plating)
B
Dividing
Quiescent
p27
Actin
WT+vector ATG5-/- WT+vector ATG5-/-

Figure S2

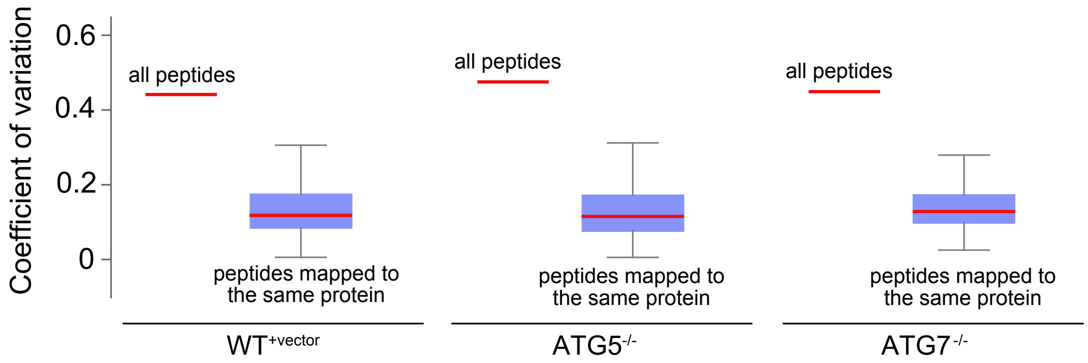
Coefficient of variation
0.6
0.4
0.2
0
all peptides
peptides mapped to the same protein
all peptides
peptides mapped to the same protein
all peptides
peptides mapped to the same protein
WT+vector
ATG5-/-
ATG7-/-

Figure S3

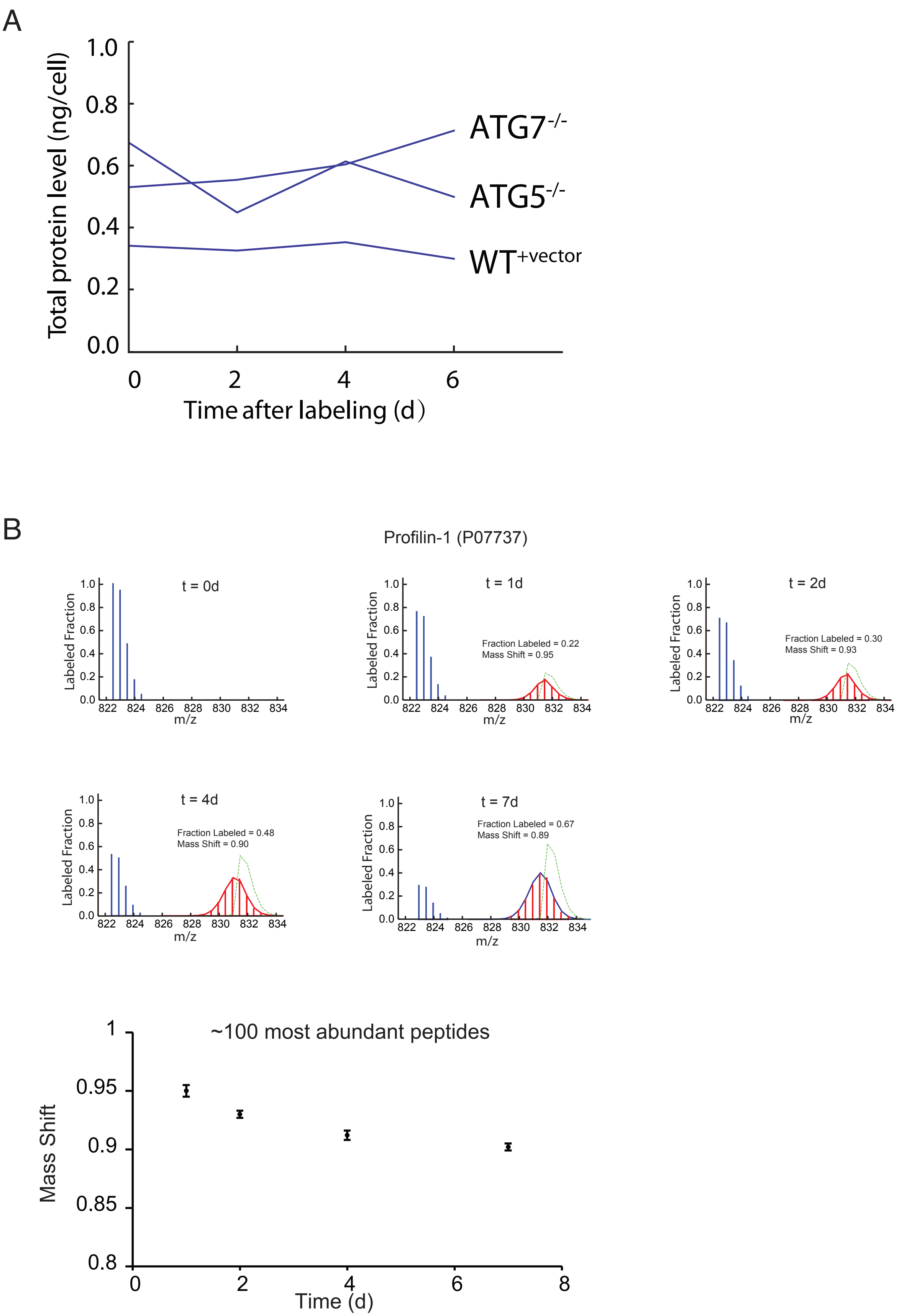
A
Total protein level (ng/cell)
1.0
0.8
0.6
0.4
0.2
0.0
0
2
4
6
Time after labeling (d)
ATG7-/-
ATG5-/-
WT+vector
B
Profilin-1 (P07737)
t = 0d
t = 1d
t = 2d
t = 4d
t = 7d
Labeled Fraction
m/z
822 824 826 828 830 832 834
Fraction Labeled = 0.22
Mass Shift = 0.95
Fraction Labeled = 0.30
Mass Shift = 0.93
Fraction Labeled = 0.48
Mass Shift = 0.90
Fraction Labeled = 0.67
Mass Shift = 0.89
~100 most abundant peptides
Mass Shift
1
0.95
0.9
0.85
0.8
0
2
4
6
8
Time (d)

Figure S4

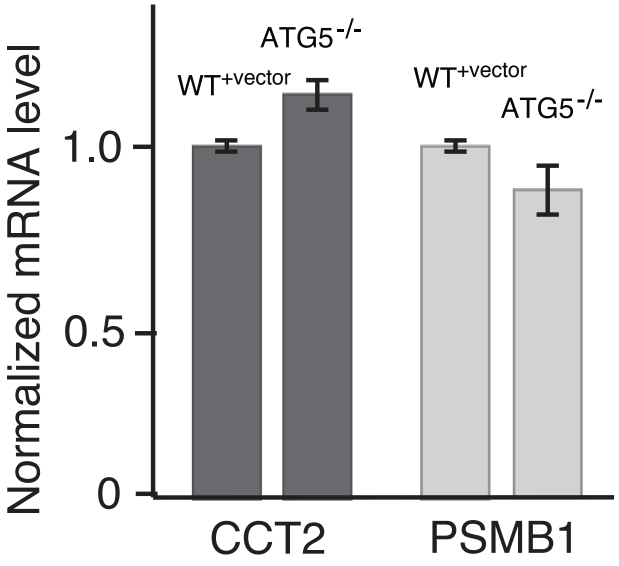
Normalized mRNA level
1.0
0.5
0
WT+vector
ATG5-/-
WT+vector
ATG5-/-
CCT2
PSMB1

Figure S5

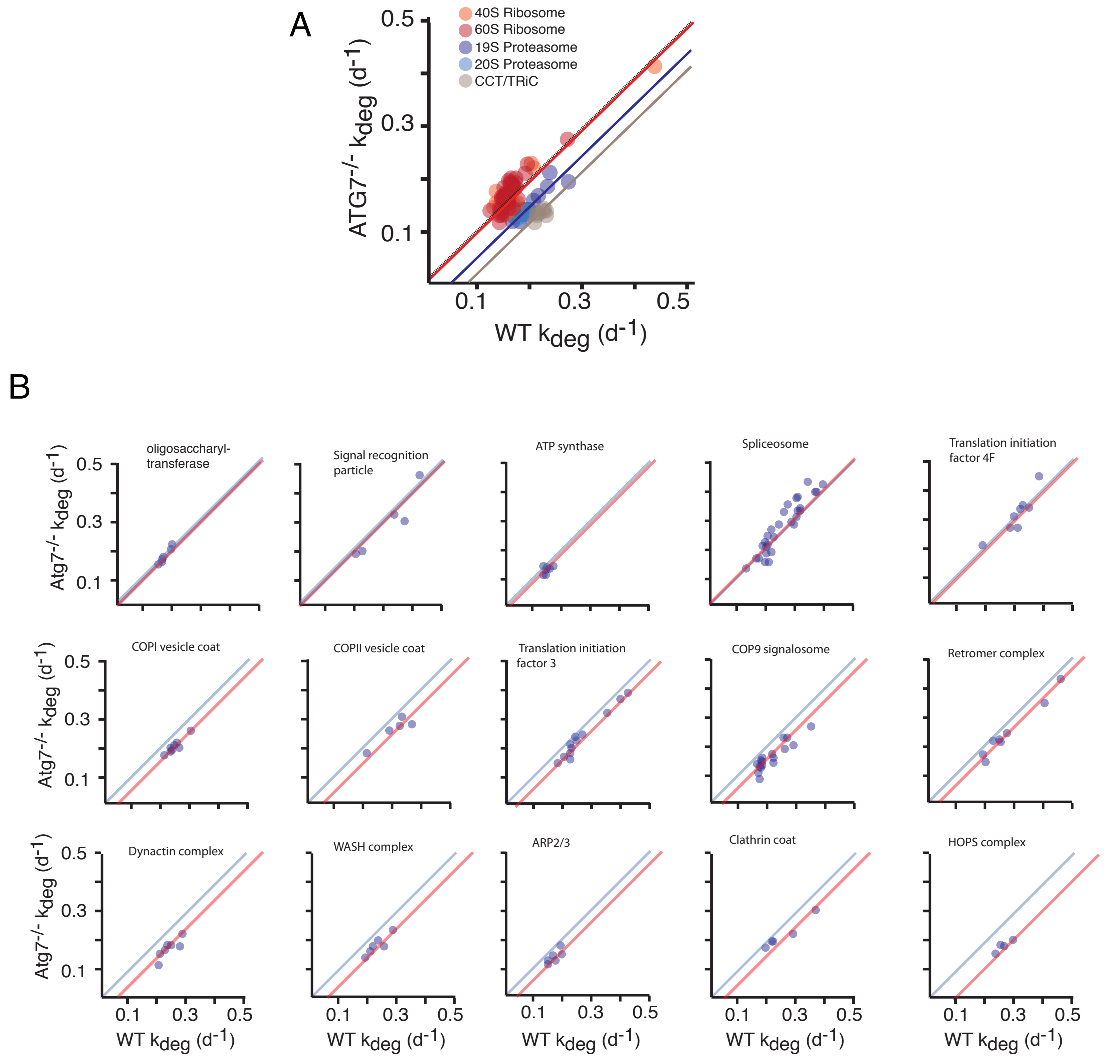
A
40S Ribosome
60S Ribosome
19S Proteasome
20S Proteasome
CCT/TRiC
ATG7-/- kdeg (d-1)
WT kdeg (d-1)
0.1
0.3
0.5
B
oligosaccharyl-transferase
Signal recognition particle
ATP synthase
Spliceosome
Translation initiation factor 4F
COPI vesicle coat
COPII vesicle coat
Translation initiation factor 3
COP9 signalosome
Retromer complex
Dynactin complex
WASH complex
ARP2/3
Clathrin coat
HOPS complex
Atg7-/- kdeg (d-1)
WT kdeg (d-1)

Figure S6
